# Vaccination of Immunocompromised Cats

**DOI:** 10.3390/v14050923

**Published:** 2022-04-28

**Authors:** Katrin Hartmann, Karin Möstl, Albert Lloret, Etienne Thiry, Diane D. Addie, Sándor Belák, Corine Boucraut-Baralon, Herman Egberink, Tadeusz Frymus, Regina Hofmann-Lehmann, Hans Lutz, Fulvio Marsilio, Maria Grazia Pennisi, Séverine Tasker, Uwe Truyen, Margaret J. Hosie

**Affiliations:** 1 Clinic of Small Animal Medicine, Centre for Clinical Veterinary Medicine, LMU Munich, 80539 Munich, Germany; 2 Institute of Virology, Department for Pathobiology, University of Veterinary Medicine, 1210 Vienna, Austria; karinmoestl@gmail.com; 3 Fundació Hospital Clínic Veterinari, Universitat Autònoma de Barcelona, Bellaterra, 08193 Barcelona, Spain; albert.lloret@uab.es; 4 Veterinary Virology and Animal Viral Diseases, Department of Infectious and Parasitic Diseases, FARAH Research Centre, Faculty of Veterinary Medicine, Liège University, 4000 Liège, Belgium; etienne.thiry@ulg.ac.be; 5 Veterinary Diagnostic Services, School of Veterinary Medicine, College of Medical, Veterinary and Life Sciences, University of Glasgow, Glasgow G61 1QH, UK; draddie@catvirus.com; 6 Department of Biomedical Sciences and Veterinary Public Health (BVF), Swedish University of Agricultural Sciences (SLU), 750 07 Uppsala, Sweden; sandor.belak@slu.se; 7 Scanelis Laboratory, 31770 Colomiers, France; corine.boucraut@scanelis.com; 8 Department of Biomolecular Health Sciences, Faculty of Veterinary Medicine, University of Utrecht, 3584 CL Utrecht, The Netherlands; h.f.egberink@uu.nl; 9 Department of Small Animal Diseases with Clinic, Institute of Veterinary Medicine, Warsaw University of Life Sciences-SGGW, 02-787 Warsaw, Poland; tadeusz_frymus@sggw.edu.pl; 10 Clinical Laboratory, Department of Clinical Diagnostics and Services, Vetsuisse Faculty, University of Zurich, 8057 Zurich, Switzerland; rhofmann@vetclinics.uzh.ch (R.H.-L.); hans.lutz@uzh.ch (H.L.); 11 Faculty of Veterinary Medicine, Università Degli Studi di Teramo, 64100 Teramo, Italy; fmarsilio@unite.it; 12 Dipartimento di Scienze Veterinarie, Università di Messina, 98168 Messina, Italy; mariagrazia.pennisi@unime.it; 13 Bristol Veterinary School, University of Bristol, Bristol BS40 5DU, UK; s.tasker@bristol.ac.uk; 14 Linnaeus Veterinary Ltd., Shirley, Solihull B90 4BN, UK; 15 Institute of Animal Hygiene and Veterinary Public Health, University of Leipzig, 04103 Leipzig, Germany; truyen@vetmed.uni-leipzig.de; 16 MRC—University of Glasgow Centre for Virus Research, Glasgow G61 1QH, UK; margaret.hosie@glasgow.ac.uk

**Keywords:** feline, immunocompromise, immunosuppression, immunodeficiency, vaccine, efficacy, safety, duration of immunity, DOI

## Abstract

Immunocompromise is a common condition in cats, especially due to widespread infections with immunosuppressive viruses, such as feline immunodeficiency virus (FIV) and feline leukaemia virus (FeLV), but also due to chronic non-infectious diseases, such as tumours, diabetes mellitus, and chronic kidney disease, as well as treatment with immunosuppressive drugs, such as glucocorticoids, cyclosporins, or tumour chemotherapy. In this review, the European Advisory Board on Cat Diseases (ABCD), a scientifically independent board of experts in feline medicine from eleven European countries, discusses the current knowledge and rationale for vaccination of immunocompromised cats. So far, there are few data available on vaccination of immunocompromised cats, and sometimes studies produce controversial results. Thus, this guideline summarizes the available scientific studies and fills in the gaps with expert opinion, where scientific studies are missing. Ultimately, this review aims to help veterinarians with their decision-making in how best to vaccinate immunocompromised cats.

## 1. Introduction

Vaccination of immunocompromised individuals is an important issue, not only in human medicine, but also in veterinary medicine, and especially in cats. There are a number of immunocompromising conditions in cats, due to common infectious diseases with immunosuppressive viruses, such as feline immunodeficiency virus (FIV) and feline leukaemia virus (FeLV), and to chronic non-infectious immunocompromising diseases, such as tumours, diabetes mellitus, and chronic kidney disease, as well as treatment with immunosuppressive drugs, such as glucocorticoids, cyclosporins, or tumour chemotherapy. The European Advisory Board on Cat Diseases (ABCD), a scientifically independent board of experts in feline medicine from eleven European countries, created this review based on their guidelines on “vaccination of immunocompromised cats” [1] to help veterinarians with decision making in how to vaccinate immunocompromised cats. Boxes at the end of each chapter are intended to give a summary for the reader and a consensus expert recommendation based on the data available. In this review, the terms immunocompromised and immunocompromise are generally used and are considered alternative terms for immunosuppressed and immunosuppression, as well as immunodeficient and immunodeficiency. Immunocompromise is a reduction of the activation or efficacy of the immune system. An animal or person who is undergoing immunosuppression or whose immune system is weak for other reasons is classified as immunocompromised or having an immunocompromised condition. An immunosuppressant is any agent that weakens the immune system, including infectious agents, immunosuppressive drugs and toxins. Immunodeficiency (or immune deficiency) is the state resulting from immunocompromise in which the immune system’s ability to combat infectious diseases and tumours is compromised or completely absent.

Life expectancy in cats has been increasing in recent decades, especially in privately owned cats receiving good preventive, medical and nutritional care. Simultaneously, the prevalence of chronic diseases increases in older animals. Now, senior cats represent a large percentage of patients in practice; this tendency will probably even increase further in the future. In human medicine, specific recommendations exist for the vaccination of immunocompromised people, such as the “Recommendations of the Advisory Committee on Immunization Practices (ACIP): Use of Vaccines and Immune Globulins in Persons with Altered Immunocompetence” of the Centers for Disease Control and Prevention (CDC) [2] or the Infectious Diseases Society of America (IDSA) Clinical Practice Guideline for Vaccination of the Immunocompromised Host [3], as well as several review articles [4,5,6]. In addition, comprehensive systematic reviews and meta-analyses on specific vaccinations, for example influenza vaccination in immunocompromised people, have been published [7,8,9], and since vaccines against SARS-CoV-2 have become available, recommendations also exist for COVID-19 vaccination in immunocompromised people [10,11,12]. Data in cats are limited and not many controlled studies in immunocompromised animals have been conducted. Thus, many of the following recommendations are based on data obtained either in humans or dogs, as well as on expert opinions.

The degree to which an individual cat is immunocompromised should be determined by the veterinarian. Severe immunocompromise can be due to a variety of conditions, including congenital immunodeficiency, FIV or FeLV infection, tumours, tumour chemotherapy or radiation, glucocorticoids, cyclosporins, or other immunosuppressive drugs. For some of these conditions, the affected cats will be severely immunocompromised; for others, such as FIV infection, the spectrum of different infection stages will determine the degree to which the immune system is compromised. As a general recommendation, cats with acute diseases or undergoing short-term immunosuppressive treatment should not be vaccinated, and vaccination should be postponed until recovery or after termination of the treatment course. However, in some situations, postponing vaccination would pose a significant risk for the cat, such as when entering a shelter environment with high infectious pressure. In these specific situations, vaccination might be necessary despite acute illness or poor general condition. For sick cats, any decision about vaccination must be taken for the individual cat, but when entering a shelter, vaccination is recommended whenever and as soon as justifiable [13]. In acutely ill cats when immediate protection (against infectious diseases) is required, passive immunisation should be used instead of active immunisation (e.g., with serum containing antibodies against feline panleukopenia virus (FPV), feline herpesvirus (FHV), and feline calicivirus (FCV), as a commercial compound or as hyperimmune serum obtained from another cat).

Some cats are immunocompromised long-term, and this review will concentrate on these conditions. Some important points must be considered when vaccinating immunocompromised cats, including

(1) the safety of modified-live virus vaccines and the concern that the infectious agent in certain vaccines might regain its pathogenicity if the immune system is not working properly,
(2) the question of whether vaccines work at all in immunocompromised cats and whether the duration of immunity after vaccination is shortened compared to healthy cats,
(3) the concern that in some of these conditions, e.g., in cats with FIV infection or chronic kidney disease, vaccination and resulting immunostimulation might lead to a progression of the disease.

## 2. Cats with Congenital Immunodeficiency Disorders

Congenital (primary) immunodeficiency in cats has rarely been described [14,15,16]. In human medicine, it is recommended that patients with primary immunodeficiency should receive all routine vaccines based on the CDC’s schedule. None of the vaccines are contraindicated [3]. Due to the lack of data in cats, this recommendation should be followed in cats with congenital immunodeficiency as well.

In conclusion, as for **cats with congenital 
immunodeficiency disorder** no data are available, these cats should be 
vaccinated as clinically healthy cats.

## 3. Cats with Retrovirus Infections

In domestic cats, three retroviruses have been identified: FIV, FeLV, and feline foamy virus (FeFV). All three are global and widespread, but differ in their potential to cause disease [17]. FeFV (previously known as feline syncytium-forming virus, FeSFV), a spumavirus, is not associated with disease [18], and no special management is required in cats with FeSFV infection. FIV, a lentivirus that shares many properties with human immunodeficiency virus (HIV), can cause an acquired immunodeficiency syndrome in cats, leading to increased risk for opportunistic infections, neurologic diseases, and tumours. In the majority of naturally infected cats, FIV infection does not cause severe clinical syndromes, and with proper care, FIV-infected cats can live for many years [19,20,21]. FeLV, an oncornavirus, is the most pathogenic of the three viruses. Even though progressive FeLV infection is associated with a decrease in life expectancy, many FeLV-infected cats kept solely indoors will live for many years with good quality of life [22]. Cats with FIV or FeLV infection can have long asymptomatic stages with no or only little immunocompromise, but at later stages can become severely immunocompromised.

### 3.1. Cats with Feline Immunodeficiency Virus (FIV) Infection

FIV infection leads to progressive disruption of normal immune function [19,23]. Early and persistent immunologic abnormalities that occur after experimental [24,25] and natural [26,27] infection include decreases in both the number and relative proportions of CD4^+^ T cells in the peripheral blood as well as in lymphoid tissues. Ultimately, loss of CD4^+^ T cells impairs immune responses because CD4^+^ T cells play critical roles in promoting and maintaining both humoral and cell-mediated immunity. Over time, lymphocytes lose the ability to proliferate in response to stimulation with lymphocyte mitogens or recall antigens, and have impaired priming by immunogens in vitro [28,29,30,31,32,33,34]. Lymphocyte function can also be impaired by reduced or altered expression of cell surface molecules, such as CD4^+^, major histocompatibility complex antigens or other co-stimulatory molecules, cytokines and cytokine receptors [35,36,37], or even the expression of abnormal molecules [38]. Many of these molecules have a critical role in antigen presentation or amplification and control of immune responses.

It has been proposed that cats with FIV infection should solely receive inactivated vaccines, if possible, out of the concern that the virus components of modified-live virus vaccines given to immunocompromised animals might regain pathogenicity [39,40,41]. However, there is no definitive scientific proof that FIV-infected cats are at increased risk from modified-live virus vaccines [42]. The use of “old” modified-live virus vaccine against canine distemper virus (CDV) in dogs experimentally infected with canine parvovirus (CPV) resulted in the development of encephalomyelitis, which was presumably due to an immunosuppressive effect of CPV infection. It has been reported that FIV-infected cats have developed illness after being vaccinated with modified-live panleukopenia vaccine [43]. However, in a pilot study comparing reaction to vaccination of four asymptomatic FIV-infected cats after modified-live panleukopenia vaccination to that of uninfected healthy cats in the field, no vaccine-associated adverse events (VAAEs) were noted [44].

The efficacy of vaccination seems to depend on the stage of FIV infection. It has been shown that FIV-infected cats in an early stage of infection are able to mount appropriate levels of protective antibodies after vaccination [44,45], but responses can be impaired during the terminal phase of infection [46]. One pilot study compared the immune response of four asymptomatic FIV-infected cats within a period of 28 days after modified-live virus vaccination against FPV to that of not-infected healthy cats in the field; there were no differences in antibody levels between the two groups and none of the FIV-infected cats developed illness or VAEEs [44]. In another study, 15 cats experimentally infected with FIV, and 15 FIV-negative control cats, received a FeLV vaccine. High anti-FeLV-antibody titres developed after vaccination in both FIV-infected and FIV-negative cats. After challenge with FeLV, FIV-infected cats were protected as well as the non-FIV-infected cats. Thus, in this study, at least in the early stage of FIV infection, the immune system was not markedly compromised and, therefore, cats could be successfully immunized [47]. In a follow-up experimental study, long-term protection of a FeLV vaccine in FIV-infected cats was determined following 30 cats for over three years. Half of the cats had previously been infected with FIV; the other 15 animals served as non-infected controls. There was no difference in the efficacy of the FeLV vaccine between FIV-infected and FIV-negative cats. After three years of observation, the FeLV-vaccinated FIV-infected cats had significantly higher survival rates as well as better clinical and laboratory parameters than the not-FeLV-vaccinated FIV-infected animals, thus indicating that the FeLV vaccine was effective in these FIV-infected cats [48]. In contrast, in one study investigating the effect of experimental primary-stage FIV infection on feline calicivirus (FCV) vaccination and subsequent challenge, there was a difference between FIV-infected and non-infected cats. Although there was some level of protection through vaccination, clinical signs of acute FCV-associated disease were more widespread in the cats infected with FIV than in those which were non-infected. FIV infection also prolonged the shedding of FCV, with more FIV-infected cats becoming chronic carriers. There was also evidence of an impaired anti-FCV-neutralizing antibody response in FIV-infected cats following FCV challenge [49]. In addition, in a five-year field trial aimed to control FeLV infection by vaccination in a colony of 30 adult cats naturally exposed to FeLV, FeLV vaccination was effective in FIV-uninfected cats, but failed to protect FIV-infected cats against FeLV [50]. Obviously, there are major differences in the response to vaccination depending on the immune status of the individual FIV-infected cat.

In addition to concerns about efficacy, there is debate about the negative effects of vaccine-induced immunostimulation in FIV-infected cats, as immunostimulation could potentially lead to progression of FIV infection by altering the balance between the immune system and the virus [23]. Although some studies even suggest that immunostimulation can help to stabilize CD4^+^ T cell numbers [51], vaccination of chronically infected FIV-infected cats with a synthetic peptide on the other hand was associated with a decrease in the CD4^+^/CD8^+^ ratio [52]. Stimulation of FIV-infected lymphocytes is known to promote FIV production in vitro, and in vivo, lymphocyte stimulation can increase the expression of cellular FIV receptors and increase virus production, a combination that could enhance progression of infection. Thus, vaccination and antigenic stimulation might potentially be disadvantageous.

In conclusion, if adult **FIV-infected cats** that 
had been vaccinated previously are kept strictly indoors, the risk of being 
infected with other pathogens is likely lower than the possible harmful effect 
of vaccination. Ideally, antibody levels against FPV should be determined and 
FPV vaccination should only be considered in cats lacking protective 
antibodies. If antibody measurement is not possible, booster vaccinations in 
adult indoor-only cats, that have received previous vaccinations, are not 
recommended. If potential exposure to FPV, FHV, or FCV cannot be excluded, only 
core vaccines should be administered, preferably in an inactivated form.

### 3.2. Cats with Feline Leukaemia Virus (FeLV) Infection

Cats with progressive FeLV infection are more severely immunocompromised than those with FIV infection [17,22,53,54]; they have suppressed cellular and humoral immunity, thus predisposing them for just about any type of infection. Therefore, maintaining a good level of protection is considered very important. While FIV preferentially replicates in CD4^+^ T cells and macrophages, FeLV can replicate and destroy virtually all haematopoietic cells. Lymphopenia and neutropenia are common in FeLV-infected cats. In some cats, lymphopenia is characterized by the preferential loss of CD4^+^ T cells, resulting in an inverted CD4^+^/CD8^+^ ratio (comparable to FIV infection) [55,56], but more commonly, substantial losses of both CD4^+^ and CD8^+^ T cells occur [56]. Many immune function tests of naturally FeLV-infected cats are abnormal, including poor response to T-cell mitogens, prolonged allograft reaction, reduced immunoglobulin production, depressed neutrophil function, complement depletion, and altered cytokine levels [57]. Finally, primary and secondary humoral responses to specific antigens are delayed and decreased in FeLV-infected cats. Cats with FeLV-associated myelosuppression have a particularly strong immunosuppression because of the occurring pancytopenia [53].

Although it has been recommended that FeLV-infected cats should preferably receive inactivated vaccines and not those containing modified-live virus (when available), there is little evidence that such cats are indeed at increased risk of VAAEs through those vaccines [58]. One pilot study comparing the response to vaccination in four asymptomatic FeLV-infected cats after modified-live virus vaccination against FPV to that of non-infected healthy cats in the field did not detect any illness or VAAEs in any of the FeLV-infected cats [44].

It has been shown that cats with progressive FeLV infection might not adequately respond to vaccination. When cats with FeLV infection were vaccinated with rabies vaccines, they were only protected for six months [59]. This has been proven only for rabies but might also be true for other vaccine components. In contrast, one pilot study that compared the immune response of four asymptomatic FeLV-infected cats after modified-live virus vaccination against FPV to that of non-infected healthy cats in the field observed no differences in antibody levels between the two groups. However, in this study, evaluation of the immune response was not performed beyond 28 days after vaccination [44].

In conclusion, for good protection of **FeLV-infected 
cats**, vaccination with core vaccines (against FPV, FHV, and FCV) should be 
performed regularly, even if the cat is kept strictly indoors (this is 
different to FIV-infected cats). If an owner cannot be convinced to keep a cat 
with progressive FeLV infection inside, rabies vaccinations can be given (in 
accordance with state and local regulations). Protection in a FeLV-infected cat 
after vaccination might not be as complete and long-lasting as in an uninfected 
cat. Thus, either more frequent vaccinations (e.g., annually), or measurement 
of anti-FPV-antibody titres to ensure sufficient protection against FPV is 
recommended for FeLV-infected cats.

## 4. Cats with Tumours

Patients with neoplastic conditions can be immunocompromised for several reasons, including conditions caused by the tumour itself, e.g., debilitation, acquired disorders of antibody production and cell-mediated immunity, and the drugs used to treat the tumour [60]. Furthermore, active tumour growth is associated with profound protein loss, which can also impair the immune response [41]. Splenectomy performed to remove a splenic tumour can also compromise the patient [61]. Tumours can lead to immunosuppression that favours tumour progression and metastasis and evolves by constitution of an immunosuppressive network, which is mediated by several tumour-derived soluble factors, such as interleukin-10, transforming growth factor-β, and vascular endothelial growth factor, and which extends from the primary tumour site to secondary lymphoid organs and peripheral vessels [62]. In this context, the tumour microenvironment is of importance [63]. Tumour-associated macrophages can be attracted by the production of chemokines from mesenchymal stromal/stem cells within the tumour [64]. Interactions between tumours and the immune system are very complex, and further factors leading to immunocompromise are being discovered. For example, a specific suppression of the secretion of interferon-γ was detected in cultured splenocytes of mice with murine breast cancer mediated by a soluble protein [65].

The nature of immunocompromise can vary depending on the type of tumour. Some specific tumours, such as multiple myeloma and some lymphomas, can cause acquired disorders of antibody production. This is more likely to happen when the tumour cells produce a paraprotein increasing globulin production but simultaneously interfering with the patient’s normal antibody response [61,64,66]. In cats with tumour-associated disorders of antibody production, vaccination is very unlikely to be effective. There are also neoplastic disorders that can cause neutropenia, which is amongst the most important risk factors for serious infection in the immunocompromised host. A severe neutropenia can be seen in myelophthisic disease caused by the spread of the tumour to the bone marrow. Myelophthisis can occur with both lymphoma and carcinoma types of neoplasia [61].

In humans, meta-analyses on the efficacy of influenza vaccination in patients with tumours revealed a significantly reduced immunological response in patients with tumours compared to controls, although this was not the case in all studies. Adult human tumour patients had depressed antibody responses to immunisation even before starting chemotherapy [67]. On the other hand, no evidence of serious VAAEs or disease progression was identified as being related to the administration of influenza vaccine. Thus, recommendations in human medicine state that vaccination should be maintained in humans with tumours [3,9], but no modified-live virus vaccines should be administered, because replication of the vaccine virus could be enhanced in severely immunocompromised persons [68,69].

A few studies in dogs demonstrated immunosuppression associated with various tumours, such as lymphoma [70,71,72,73]. Dogs with lymphoma had reduced T cell numbers when compared to healthy dogs, and dogs with osteosarcoma also had reduced T and B cell numbers [74]. One study demonstrated the immunosuppressive network in dogs with mammary carcinoma; while the number of various T cell subpopulations was constant during tumour development, the number of regulatory T cells was significantly higher in tumour-bearing dogs than in healthy individuals, as was the number of myeloid-derived suppressor cells [75]. This heterogeneous cell population in mostly immature developmental stages induces molecules and factors that are essential for tumour growth and neo vascularization; however, they also have a potent inhibitory activity on tumour-specific as well as non-specific T cells and, thus, contribute significantly to the dysfunction of the T cell-mediated immune response [76]. In one study, dogs with lymphoma or osteosarcoma were vaccinated, and post-vaccination antibody titres were compared to those of a healthy control group. Although dogs with lymphoma or osteosarcoma appeared to be relatively T cell-deficient, antibody titres after vaccination were not significantly different to those of healthy controls [74].

No studies have been performed in cats with tumours to demonstrate their ability to react to vaccination. However, one study assessed the prevalence of antibodies against FPV in 350 client-owned cats and identified factors that were associated with a lack of antibodies in cats. Factors including information regarding signalment, origin, environment, lifestyle, housing conditions, health status, chronic diseases, glucocorticoid therapy, and vaccination status were analysed by multivariable logistic regression analysis. Of the 350 cats, 103 (29.4%) had no antibodies against FPV and, among other factors, tumours were significantly associated with a lack of antibodies [77]. Thus, in cats with tumours, the protection rate is not comparable to that in healthy cats.

In conclusion, in **cats with tumours**, antibody 
measurement against FPV infection is a good option to confirm protection 
against panleukopenia. Vaccination can be considered in otherwise healthy cats 
with tumours not receiving chemotherapy. In those receiving chemotherapy, 
vaccination ideally should precede the initiation of chemotherapy by at least 
two weeks. If this is not possible, vaccination should be postponed until at 
least three months after the end of chemotherapy.

## 5. Cats with Other Immunosuppressive Diseases

There are several other diseases that can alter the immune system, such as diabetes, chronic kidney disease, and asplenia. In humans, these conditions increase the patient’s risk for certain diseases, and thus, specific vaccines, particularly bacterial vaccines, are recommended for such patients [3,78,79]. Frequently, the immune response of those patients to bacterial antigens is not as good as that of immunocompetent people, and higher doses or more frequent boosters might be required. In humans, liver cirrhosis is also included in the guidelines as an important immunocompromising disease [3]; this is a very rare condition in cats and thus will not be further discussed.

### 5.1. Diabetes Mellitus

Diabetes mellitus can alter the body’s immune defences, therefore rendering the patient predisposed to infection. The reasons for this have not been completely explained but can involve abnormalities with cell-mediated immunity [80] and abnormal phagocyte function [81], as well as poor blood supply to various body tissues because of diabetic vascular disease [61]. Thus, infections in animals with diabetes are more common and severe and can involve the skin, urinary tract, and other body sites, such as the gall bladder and liver [61]. In diabetic cats, urinary tract infections are the most common secondary infections [82,83].

Although several in vitro tests of immunologic function are known to be abnormal among diabetic patients, these defects are likely of little clinical importance. In humans with longstanding diabetes, who often have cardiovascular, renal, and other end-organ dysfunctions, vaccinations are recommended. In one study, patients receiving either insulin or oral antidiabetic medications responded normally to influenza vaccination without impairment of diabetic control [84]. Also, pneumococcal vaccines were safe and effective in diabetic patients and did not interfere with insulin levels or diabetic control [85,86]. In a study on the vaccination of elderly people, patients with diabetes showed an immune response comparable to that of other non-diabetic participants [87]. Still, the Advisory Committee on Immunization Practices (ACIP) of the Centers for Disease Control and Prevention recommends that adult diabetic patients should be vaccinated as early as possible after their diagnosis [2].

The immune function of a diabetic patient, however, is more severely compromised if the patient remains uncontrollably hyperglycaemic [88]. Thus, vaccinations should never be given to a cat with poorly controlled diabetes, and control of the diabetic situation should be achieved before vaccination. In cats, infections play an important role in inducing insulin resistance and by causing diabetic decompensation because of the endogenous hypersecretion of stress hormones, such as cortisol [61]. There are no data, however, that indicate that vaccination could promote diabetic decompensation.

Thus, in conclusion, the recommendation would be to 
vaccinate **diabetic cats** according to the proposed guidelines for healthy 
cats, but to postpone vaccination in an uncontrolled diabetic case until 
control is achieved. Antibody measurement against FPV infection would also be 
advised to confirm protection against panleukopenia.

### 5.2. Chronic Kidney Disease

Patients with kidney disease have an increased risk of infection with a variety of pathogens [89,90,91,92,93]. An association between chronic kidney disease and reduced antibody development following vaccination has been described in humans. The efficacy of pneumococcal vaccination for some of these patients, including those on dialysis, was considerably reduced [93,94], and their antibody levels might also be lower [95]. Therefore, they might require repeated vaccinations [96,97] or an increased number of vaccine doses. It has been shown that the stage of the kidney disease and thus the impairment of the glomerular filtration rates predict the ability to produce antibodies [98], since a rise of antibody titres after vaccination became increasingly unlikely as the glomerular filtration rate decreased [98]. Malnutrition in patients with chronic kidney disease was also suspected to be associated with an impaired immune response [99], and chronic uraemia, directly or indirectly, was shown to alter immune cell function [100]. Consequently, generalized immunosuppression and decreased antibody development are expected in chronic kidney disease patients with secondary antibody responses being less affected than primary antibody responses. Thus, immunisation strategies and especially vaccination with novel antigens should be given as early as possible during the course of chronic kidney disease [2].

No studies have been performed in cats with chronic kidney disease to demonstrate their ability to respond to vaccination. However, one study assessed the prevalence of antibodies against FPV in cats in Southern Germany and identified factors that were associated with a lack of antibodies in 350 client-owned cats, and the presence of chronic kidney disease was significantly associated with a lack of antibodies [77]. Thus, in cats with chronic kidney disease, the protection rate is not comparable to that of healthy animals.

There is another concern that should be discussed when considering vaccination in cats with chronic kidney disease. Some studies suggest a risk association between chronic kidney disease and frequent vaccination in cats [101,102]. A risk factor analysis on the development of chronic kidney disease in cats evaluated clinical and questionnaire data to identify risk factors in 148 client-owned older cats (>nine years) followed longitudinally for variable time periods. Besides dental diseases, the only significant risk factor identified in the final multivariable Cox regression model was annual/frequent vaccination, suggesting an association between frequent vaccinations and development of chronic kidney disease [101]. Such an association has also been proposed in studies that identified antibodies against feline kidney cells in vaccinated cats. Vaccine viruses are usually grown on Crandell-Rees feline kidney (CRFK) cells, and it was hypothesized that vaccinated cats would produce antibodies against CRFK cells that could interact with their own kidney tissues and, thus, could be a trigger for interstitial nephritis. Parenteral administration of CRFK cell lysates or FPV, FHV, and FCV vaccines grown on CRFK cells induced antibodies in cats against CRFK cells. These antibodies also reacted with feline renal cell extracts. In contrast, control cats that had received an intranasal vaccine did not develop detectable antibodies against CRFK cells [103]. A follow-up study tested whether interstitial nephritis could be detected in cats that were immunologically sensitized with CRFK lysates, boosted with CRFK lysates, and then had kidney biopsy two weeks after the booster. Cats were immunologically sensitized against CRFK lysates twelve times in the first 50 weeks over two years. Half of the cats sensitized with CRFK lysates indeed developed lymphocytic-plasmacytic interstitial nephritis [104]. In another study, 44 kittens were inoculated with CRFK lysates and FPV, FHV, and FCV vaccines. Several CRFK antigens were identified in the kittens, and protein isolation and sequencing identified them as alpha-enolase, annexin A2, and macrophage capping protein (MCP). Sera from vaccinated and CRFK-inoculated kittens were shown to recognize annexin A2 and alpha-enolase by western blot and indirect ELISA. In humans, alpha-enolase antibodies are nephritogenic; alpha-enolase and annexin A2 antibodies have been associated with autoimmune diseases [105], and it was shown that alpha-enolase decreases in damaged renal tubules and increases in the glomeruli of older cats prior to the development of detectable chronic kidney disease [106]. One study produced anti-cat kidney antibodies in rabbits [107] to examine serum samples and kidneys collected from 156 live and 26 cats at necropsy to evaluate whether FHV, FCV, and FPV vaccines, prepared from viruses grown in CRFK cells, could induce antibodies to cross-react with feline kidney tissues. The prevalence of autoantibodies that bound to kidney tissues in cats were 41% and 13% by ELISA and immunofluorescence, respectively; there was no direct link between vaccination and anti-kidney antibodies, but the presence of antibodies to kidney tissues was significantly associated with anti-FHV/-FCV/-FPV antibodies [108]. Although these studies suggest a possible association between vaccination and chronic kidney disease in cats, there is no causative evidence, and further studies are required.

In conclusion, however, as most of the **cats with 
chronic kidney disease** are of older age and are likely to have received 
vaccinations in the past, the risk for such a cat to acquire infectious 
diseases is considered low, and so vaccination might not be necessary. Ideally, 
antibody levels against FPV should be determined; only cats lacking protective 
antibodies should be vaccinated against panleukopenia. If antibody measurement 
is not possible, booster vaccination is not recommended for a cat with chronic 
kidney disease that has been vaccinated previously and is kept strictly 
indoors. If potential exposure to FPV, FHV, or FCV cannot be excluded, an intranasal 
vaccine should be given, if available.

### 5.3. Asplenia

People who have anatomic or functional asplenia have an increased risk for infectious diseases, especially fulminant bacteraemia associated with high mortality. It has been shown that antibodies after pneumococcal vaccination decline almost linearly by 24 to 32% within the first year after splenectomy [109]. Thus, in human medicine, vaccines, especially against bacterial pathogens, such as pneumococcal and meningococcal vaccines, are considered important for all asplenic persons [2].

Asplenia is rare in cats and mainly occurs after iatrogenic removal of the spleen. Asplenia is more common in dogs, and dogs without a spleen are at increased risk of developing clinical manifestations of bacterial or parasitic infections that are otherwise usually asymptomatic, such as infections with *Mycoplasma haemocanis* [110,111,112]. In addition, novel bacterial or parasitic species have been detected in asplenic dogs, such as a new haemoplasma species ‘*Candidatus* Mycoplasma haematoparvum’ [113] or a new large *Babesia* spp. [114]. In cats, studies on the outcome following laparatomic [115,116] or laparascopic [117] splenectomy have been performed, but no increased risk for certain infections was observed in these studies. There is only one case report of an asplenic cat that had recovered from *Cytauxzoon felis* infection following treatment with the anti-theilerial drug parvaquone, but showed an increase in piroplasm parasitaemia after splenectomy [118]; thus, asplenic cats also might be predisposed for certain intracellular bacteria or parasites.

In conclusion, in **asplenic cats**, the 
vaccination protection rate might not be comparable to that of healthy cats. 
Antibody measurement against FPV infection would be an option to confirm if 
protection is present. If antibody measurement is not possible, the 
recommendation would be to vaccinate asplenic cats according to the proposed 
guidelines for healthy cats. When elective splenectomy is planned, vaccination 
should precede surgery by at least two weeks, if possible.

## 6. Cats Receiving Immunosuppressive Therapy

Immunosuppressive drugs, such as glucocorticoids, cyclosporine, or tumour chemotherapeutics, are commonly used in cats with various diseases [119,120]. If used short-term, vaccination should be postponed until at least three months after the end of the treatment, but some cats require long-term therapy.

### 6.1. Glucocorticoid Treatment

Many clinical conditions require long-term glucocorticoid treatment, and the degree of immunosuppression depends on the glucocorticoid dosage used [43]. In many immune-mediated diseases, glucocorticoids are the initial and primary drug of choice and are started mostly at high doses. The effects of such high doses of glucocorticoids on the immune system are substantial, with effects involving various components of the immune system. The effects on neutrophils include decreases in chemotaxis and margination and impairment of phagocytosis and bacterial killing, thus predisposing the patient to infections that can involve many body tissues. The effects of glucocorticoids on macrophages result in impaired chemotaxis, phagocytosis, and bactericidal activity. Macrophages will also have decreased interleukin-1 production and antigen-processing which will further predispose the animal to infection. Glucocorticoids will cause depressed lymphocyte proliferation, depressed T cell responses, impaired T cell cytotoxicity, depressed interleukin-2 production, and depressed lymphokine production. There is also an influence on immunoglobulin production [121,122]. Patients treated with high doses of glucocorticoids will be further predisposed to infection if other cytotoxic or immunosuppressive drugs are used simultaneously [61]. In humans, organ transplant recipients receiving high dose glucocorticoids are an important specific group of severely immunocompromised people, and there are specific recommendations on vaccinations for these patients [123]; however, organ transplantation is still not very commonly performed in feline medicine. Dogs treated with prednisolone (2 mg/kg q12 h) showed a decrease in the serum concentration of all immunoglobulin classes as well as lower numbers of CD4^+^ and CD8^+^ T cells and B cells [124]. One study demonstrated a significant decrease in T cells after short-term use of prednisolone (three days with a dose between 1.66 and 2.24 mg/kg q24 h) over a period of 38 days [125].

The exact dose of systemic glucocorticoids and the duration of their administration needed to suppress the immune system in an otherwise healthy cat are not well defined. The immunocompromising effects of glucocorticoid treatment vary, but many clinicians consider a dose of 2 mg/kg q24 h prednisolone as sufficiently immunocompromising to raise concerns about the safety of vaccination with modified live-virus vaccines. Glucocorticoids used in lower (but still higher than physiologic) doses also might reduce the immune response to vaccines. In human medicine, glucocorticoid therapy usually does not contraindicate administration of vaccines (not even with modified-live virus vaccines), when glucocorticoid therapy is short-term (less than two weeks), is given in only a low to moderate doses, is given as long-term alternate-day treatment with short-acting preparations, is given to maintain physiologic doses (such as replacement therapy in patients with Addison’s disease), or is only administered locally (topically to skin or eyes, by aerosol, or by intra-articular, bursal, or tendon injection) [2].

Studies on the effects of glucocorticoid therapy on vaccination response in dogs and cats show inconsistent results. One study investigated the effect of oral prednisolone on vaccination against CDV in Beagle puppies and found that doses of 1 mg/kg q24 h and 10 mg/kg q24 h prednisolone orally over a period of 21 days had no effect on the response to vaccination [126]. The use of 0.25 mg/kg dexamethasone (which corresponds to a dose of 1.25 mg/kg prednisolone) in dogs before and after the first vaccination against rabies also had no negative effects on the antibody response [127]. A study in cats found that glucocorticoid injections (three times 2.25 mg prednisolone and 0.75 mg dexamethasone intramuscularly 48 h apart) had no effect on the humoral immune response and challenge after vaccination with an inactivated combination vaccine against feline FPV, FHV, and FCV [128]. However, one study assessed the prevalence of antibodies against FPV in cats in Southern Germany and identified factors that were associated with a lack of antibodies in 350 client-owned cats. In this study, glucocorticoid treatment was significantly associated with a lack of antibodies, and cats receiving glucocorticoids for eleven weeks and longer were particularly at risk [77].

In conclusion, if possible, veterinarians should wait 
at least three months after discontinuation of **glucocorticoid therapy** 
before administering vaccines, especially modified-live virus vaccines, to cats 
who have received high-dose, systemic glucocorticoids for more than two weeks. 
If continuous long-term glucocorticoid therapy is necessary, vaccination 
schedules should be maintained, but inactivated vaccines should be applied, if 
available. On the other hand, vaccination could be safely considered in 
well-controlled cats receiving low-dose anti-inflammatory glucocorticoid 
treatment. Antibody measurement against FPV infection is recommended to confirm 
that protection against panleukopenia is present in cats treated with 
glucocorticoids.

### 6.2. Cyclosporine Treatment

Cyclosporines are commonly used in cats, such as for feline hypersensitivity dermatitis or autoimmune diseases [129]. Cyclosporine depresses lymphocyte function [130] and can interfere with cell-mediated immunity, thus compromising the host defence system against infectious agents, such as intracellular parasites [61]. In five of ten cats that had been treated with cyclosporine at a daily dose of 20 mg/kg q24 h for four weeks, an impairment of the cell-mediated immune response was demonstrated [131]. It has been shown that cats with high cyclosporine blood concentrations at the time of primary *Toxoplasma gondii* infection can be at risk of developing systemic toxoplasmosis [132,133], that cats treated with cyclosporine can develop unusual presentations of toxoplasmosis such as *Toxoplasma gondii*-associated cholecystitis [134], that latent *Toxoplasma gondii* infection can be reactivated during treatment [135], and that in some cats being treated with cyclosporine, toxoplasmosis can be fatal [136]. Cats receiving cyclosporine are also predisposed to other infections, such as systemic *Salmonella* spp. infection [137]. It has been suggested that few client-owned cats that had received cyclosporine to block renal transplant rejection had developed clinical signs of upper respiratory tract disease that might have been caused by re-activation of FHV infection. In one study, cats experimentally inoculated with FHV several months previously were administered cyclosporine (or placebo); while clinical signs of re-activated FHV infection occurred in some cats, disease was mild in most of them and did not require specific therapy. These findings suggest that the use of cyclosporine (at least in the dosage used) is unlikely to induce significant FHV-associated disease in previously infected cats [132].

One study investigated the immunosuppressive effect of cyclosporine on the ability of cats to mount an immune response following vaccination. Thirty-two healthy, immunocompetent adult cats (16 cats/group) were treated with either cyclosporine for 56 days at a dose of 24 mg/kg q24 h (more than three times the therapeutic dose) or sham-dosed. Prior to treatment, cats had an adequate antibody response to primary vaccination against FPV, FHV, FCV, FeLV, and rabies. Booster vaccination against FPV, FHV, FCV, FeLV, and rabies or novel vaccination against FIV were administered 28 days after initiation of treatment with cyclosporine. There were delays/reductions in antibody response to FHV, FeLV, and rabies in treated cats; however, adequate protection was achieved in response to all booster vaccinations. Following primary vaccination with FIV, however, control cats showed a response, but treated cats showed no antibody production. Thus, adult cats treated with high-dose cyclosporine were able to achieve adequate protection following booster vaccination, while in contrast, cats failed to mount a humoral response to a novel vaccination. This suggests that memory B cell immune responses remain intact during high-dose cyclosporine administration in cats, but that primary immune responses are impaired [138].

Thus, in conclusion, booster vaccination can be given 
to **cats receiving cyclosporine**, but novel vaccinations should be applied 
before cyclosporine treatment is initiated or postponed until at least three 
months after the end of the treatment. Measurement of FPV antibodies is a good 
option to confirm that protection against panleukopenia is present.

### 6.3. Tumour Chemotherapy

Many of the cytotoxic drugs used for anti-tumour chemotherapy inhibit cell division, and when this occurs, B and T cells are often destroyed, thus impairing the body’s ability to produce antibodies and to allow for cell-mediated immune protection. The immune system of the tumour patient will be further compromised by the concomitant use of other immunosuppressive agents, such as glucocorticoids, and any devastating effect of myelo-phthisic tumour behaviour [61].

Administration of chemotherapeutic agents to mice and humans had variable effects on different components of the immune system. For example, lymphocyte depletion in human patients undergoing chemotherapy has been reported, but the degree of lymphocyte depletion appeared to be dependent on the particular chemotherapy protocol [139,140]. Lymphocyte depletion, specifically, depletion of CD4^+^ T cells, can even persist long after the completion of chemotherapy [141,142]. Even though this is described in humans, in FeLV-infected cats with mediastinal lymphoma, chemotherapy did not cause any significant change in the CD4^+^/CD8^+^ ratio [143]. Furthermore, not all chemotherapy agents are equally immunosuppressive. Alkylating agents, such as cyclophosphamide, are particularly prone to causing immunosuppression because of their affinity for destroying rapidly dividing cells, thus impairing the B and T cell response. These effects on the immune system are made even worse by cyclophosphamide’s ability to suppress the bone marrow and cause neutropenia [61]. However, on the other hand, in humans, cyclophosphamide administered at low doses was shown to potentiate humoral immunity and decrease immunologic tolerance [144,145]. Doxorubicin and related drugs also have different effects on adaptive immune responses, with doxorubicin being immunostimulatory and preserving cell-mediated immunity in some human studies [144,146,147,148,149,150,151]. The effects of chemotherapy on the humoral immune response can also be variable. In human paediatric oncology patients, pre-existing titres to tetanus, diphtheria, and poliomyelitis were preserved throughout chemotherapy in some, but not all studies [152,153,154]. In some studies, the ability of the humoral immune system to respond to vaccination was restored within three to twelve months of completing chemotherapy [153,155,156,157].

In dogs, chemotherapy has been shown to have no effect on pre-existing antibody titres. A prospective study determined an association between tumour chemotherapy and serum CDV, CPV, and rabies virus antibody titres in tumour-bearing dogs, including 21 client-owned dogs with various tumours and 16 with lymphoma. No significant changes were detected in CDV, CPV, and rabies virus titres following chemotherapy in tumour-bearing dogs. Thus, established immunity to CDV, CPV, and rabies virus from previous vaccination was not significantly compromised by standard chemotherapy [158]. Another prospective study evaluated the effects of two common chemotherapy protocols on T and B cell numbers and humoral immune responses to *de novo* vaccination in 21 dogs with tumours (twelve with lymphoma, nine with osteosarcoma) comparing the effects of doxorubicin versus multi-drug chemotherapy. Doxorubicin treatment did not cause a significant decrease in T or B cell numbers, whereas treatment with combination chemotherapy caused a significant and persistent decrease in B cell numbers. Antibody titres after vaccination, however, were not significantly different between control and chemotherapy-receiving dogs. These findings suggest that chemotherapy might have less impact on T cell numbers and the ability to mount antibody responses in dogs with tumours than previously anticipated, and that administration of chemotherapy does not preclude the administration of vaccines [74].

Few data are available on **cats receiving tumour chemotherapy**, but studies in dogs suggest that that chemotherapy might not have a significant impact on immunologic reactions and the ability to mount antibody responses and that administration of chemotherapy does not preclude the administration of vaccines. However, based on the recommendations in humans, when tumour chemotherapy is being considered, vaccination should ideally precede the initiation of chemotherapy or immunosuppression by at least two weeks or, if not possible, be postponed until at least three months after the end of chemotherapy. If possible, vaccination during chemotherapy should be avoided because antibody responses might be suboptimal.

## 7. Cats under General Anaesthesia

Many countries regularly perform spay/neuter and release programs to control the stray cat population. Cats in such situations are commonly very difficult to handle and are released immediately after recovering from anaesthesia. Some of these programs include vaccination of cats while still under anaesthesia for spaying/neutering to facilitate handling. Although short-term immunosuppressive effects of anaesthesia and surgery have been described [159,160], there is no evidence of a clinically relevant influence on the immune response to vaccination [161]. Humoral immune response after vaccination against CDV and CPV between ten days before and three days after surgery was found to be adequate (with an at least four-fold increase in antibody titres) in 17 of 20 dogs [162]. One prospective study determined the effects of anaesthesia and surgery on antibody development after vaccination in 32 specific-pathogen-free kittens. Kittens were assigned to one of four treatment groups: neutering at seven, eight, or nine weeks of age or no neutering. All kittens were inoculated with modified-live virus vaccines against FPV, FHV, and FCV at eight, eleven, and 14 weeks of age and inactivated vaccines against rabies virus at 14 weeks of age. Antibody response of kittens neutered at the time of first vaccination (eight weeks) were not different from those of kittens neutered one week before (seven weeks) or one week after (nine weeks) the first vaccination or from those of kittens that were not neutered. Anaesthesia and neutering at or near the time of first vaccination with a modified-live virus vaccine did not impair antibody responses in kittens [163].

Thus, in conclusion, when necessary (e.g., 
trap-neuter situation) cats can be vaccinated in the perioperative period. 
However, if easily possible, vaccination should not be given during **anaesthesia 
and/or surgery or directly afterward** but should ideally be postponed.

## 8. Geriatric Cats

Advances in veterinary medicine, nutrition, and client education have increased the life expectancy for domestic cats in recent years [164,165]. In a study in the United States, approximately 20% of pet cats were eleven years of age or older [166]; and a 2017 study in the UK showed a median age of 6.2 years in cats presenting to veterinary clinics, with cats aged more than eight years representing more than 40% of feline consultations [167]. Ageing is a continuous and slow process that compromises the normal functioning of various organs and systems in both qualitative and quantitative terms [168], and has been defined as a complex process in which the individual suffers from a decline in physical condition, organ, sensory and mental function, as well as immune response [169,170]. Obviously, there is a great individual variation between biological and chronological age and how the body systems age in each individual, meaning that geriatric abnormalities or diseases can appear earlier in some cats and never appear in others. Thus, feline aging is considered a multifaceted process that results in a progressive series of life stages, from conception to senescence. It is influenced by the host’s genetics plus innumerable internal and external factors and results in the progressive decline in the ability to maintain homeostasis when challenged by physiologic and environmental stressors [166]. These factors include previous injuries, diseases, nutritional status, and environmental challenges, and thus, every cat ages slightly differently.

It has been proposed to classify older cats as mature (7–10 years), seniors (11–14 years), and geriatric or super senior (> 15 years) [171]. Seniority in animals is defined as beginning at an age that is 25% below the average life expectancy within the species/breed, which would be calculated as eleven years in cats [172]. Although feline life stage guidelines [173] and general senior care guidelines have been published by the American Association of Feline Practitioners (AAFP) [174] and the American Animal Hospital Association (AAHA) [172] and implementation of senior/geriatric health care program in veterinary practice has been proposed [175], so far, there are no specific vaccination recommendations for senior or geriatric cats, and there is a general lack of knowledge if geriatric cats have special vaccination needs.

Ageing and the geriatric decline of body systems can lead to a decrease in immune function (immunosenescence) [176,177,178,179] and also to a pro-inflammatory state (inflammageing) plus the presence of degenerative, neoplastic, or inflammatory/immune-mediated diseases, which all can have an impact on the susceptibility to infectious diseases and/or produce an abnormal or decreased response to vaccination [177,180,181,182,183,184,185].

“Immunosenescence” has been defined as a multifactorial complex of changes that occurs in the immune system of elderly individuals that predisposes them to increased morbidity and mortality to infection and age-related pathology. It has recently been suggested that immunological changes in immunosenescence resemble those observed following chronic stress or corticosteroid treatment [186]. Immunosenescence can result in a reduced ability to screen for neoplastic cells and fight infection [179], and thus render individuals more susceptible to certain infections; for example, cystitis in younger cats in less than 15% of cases is caused by bacterial infection, compared with up to 50% in cats ten years or older [187,188]. Immunosenescence could also reduce the ability to efficiently mount an immune response after vaccine administration. “Inflammageing” has been defined as the effect of a lifetime of constant antigenic challenge and associated production of inflammatory mediators that can trigger the onset of inflammatory disease in older individuals. Immunosenescence and inflammageing have also been shown to occur in cats [164,179], but there are no data about the effects of these conditions on the immune responses after vaccination.

Several immunological differences have been demonstrated in some studies in senior and geriatric cats when compared to younger adult cats, including a lower number of circulating leucocytes (lymphocytes, CD4^+^ T cells, CD8^+^ T cells, B cells, CD56^+^ NK cells, and eosinophils), elevated concentrations of IgM and IgA, lower levels of insulin-like growth factor (which can be associated to lower numbers of CD4^+^ T cells), reduced blood lymphocyte blastogenic responses to stimulation with several mitogens, and increased monocyte production of pro-inflammatory cytokines (determined by mRNA levels) [179]. These changes demonstrate some alteration of the immune response, but do not tackle all elements of immunosenescence as described in general in geriatric mammals, such as the innate immune response that might be unaltered or even increased [189], the ability to mount a primary serum antibody response to a novel antigen being unaltered despite a decrease in B cell numbers, the presence of antibodies with lower affinity [189], quicker antibody titre decrease [177], the ability to adequately retain B cell memory and serum antibody concentrations that, however, less effectively respond to primary immunization, the decrease in the CD4^+^/CD8^+^ ratio, the decreased number of naive T cells [189], and the increase in memory CD4^+^ and CD8^+^ T cells.

In humans, specific guidelines for elderly people (generally > 60 years of age) exist, and increased vulnerability to infection of the elderly makes them a particularly important target population for vaccination. Most vaccines are considered less immunogenic and efficient in elderly people because of age-related changes in the immune system. Various strategies, such as the use of specifically designed vaccines for elderly people (e.g., novel adjuvants and administration routes) have been proposed. As antibody titres are generally lower in the elderly and decline faster, regular booster vaccinations are considered essential to ensure protection [190].

In cats, to this point no studies have been published on the response of senior or geriatric cats to vaccination, and the question arises whether either immunosenescence or inflammageing might have an impact on immunity which should lead to changes in vaccination protocols in old cats. There are no data that would support the idea of infectious diseases being more common in senior or geriatric cats, and the incidence of infectious diseases preventable by vaccination in senior and geriatric cats is generally considered low. Old cats rarely die or present with signs of those infections [191,192]. On the other hand, it is also not known whether vaccine boosters could worsen a pro-inflammatory state in a senior or geriatric cat, and thus reducing the number of booster vaccinations would seem appropriate. In addition, many senior or geriatric cats are diagnosed with chronic inflammatory or immune-mediated diseases, such as chronic gingivitis or periodontal disease, chronic kidney disease, inflammatory bowel disease, inflammatory liver disease, or pancreatitis, on which frequent vaccinations might have a negative impact.

Although so far no studies have been performed on the response of senior or geriatric cats to vaccination, duration of immunity (DOI) studies have shown long-term immunity against FPV, FHV, and FCV [193,194,195], and experimental studies have demonstrated that immunity persists for years, showing that immunological memory to core vaccines is adequate, as well as the immunological response to boosters [178,196]. Based on these experimental studies and expert opinion, healthy geriatric cats properly vaccinated should receive boosters at recommended intervals based on previously published guidelines and following the assessment of individual risk [1]. On the other hand, there is some evidence that older cats might not respond efficiently to novel antigens that are administered for the first time. This has been shown with rabies vaccination in dogs but could extend to other antigens. Older dogs vaccinated for the first time against rabies showed lower antibody levels compared to younger dogs, in general with titres below 0.5 IU [197]. In addition, it is known that the number of naive T cells decreases with increasing age, also resulting in an impaired immune response to new antigens [179,185,198].

Thus, in conclusion, based on these findings, if 
healthy **senior or geriatric cats** need to be vaccinated against a novel pathogen for the first time (e.g., travelling, moving, changing life style), even if the regular vaccination schedule consists of one injection (e.g., rabies), a single dose should not be considered enough to ensure proper 
immunisation, and a second dose is recommended in these animals at a three to 
four week interval. Alternatively, FPV and rabies antibodies could be measured 
after the first injection to verify that protection is adequate. Cats that need 
regular re-vaccinations should be vaccinated according to the proposed 
guidelines for healthy adult cats.

## 9. Conclusions

Immunocompromise is a common condition in cats, but so far, in contrast to human medicine, no specific guidelines were available for vaccination of immunocompromised cats. Thus, this review based on the guidelines of the European Advisory Board on Cat Diseases (ABCD) [1] was produced with the goal of helping veterinarians in their decision-making for how best to vaccinate immunocompromised cats. A summary of the recommendations is given in Table 1. The degree to which an individual cat is immunocompromised should always be determined by the veterinarian.

Generally, cats with acute disease or short-term immunosuppressive treatment should not be vaccinated, and vaccination should either be postponed until the disease is cured and/or treatment has been stopped or in the case of short-term treatment and if possible be given prior (e.g., two weeks) to the start of the immunosuppressive treatment.

In most of the chronic immunocompromising conditions (Table 1), the presence of FPV antibodies should be determined as a measure of protection and ability to mount an adequate immune response [199]. In some immunocompromising conditions, cats can be vaccinated in the same way as clinically healthy cats (such as healthy senior or geriatric cats, cats with congenital immunodeficiency disorders, or asplenic cats). Sometimes it is recommended also in chronic conditions that the vaccination should be postponed until the disease is well under control (such as in cats with tumours or diabetes) or until the treatment is paused or finished (such as in cats receiving tumour chemotherapy, high-dose glucocorticoids, or cyclosporine). In some conditions, vaccination should be avoided if possible (such as cats with chronic kidney disease or FIV infection) and those cats as well as their companion cats should be kept strictly indoors to avoid contact with infectious agents. In other conditions, however, more frequent vaccination is recommended (such as in cats with FeLV infection). This guideline will continue to be updated regularly on the ABCD homepage (www.abcdcatsvets.org accessed on 28 April 2022) as new data become available.

## Figures and Tables

**Table 1:** Vaccination recommendations in immunocompromised cats.

Condition or Situation That Could Be Associated with Immunocompromise	Measure FPV Antibodies *	Recommended Vaccination Protocol
Acute disease or short-term immunosuppressive treatment	No **	(1); alternatively: (2)
Congenital immunodeficiency disorders	Yes	(3)
Feline immunodeficiency virus (FIV) infection	Yes	(5); (6); (7)
Feline leukaemia virus (FeLV) infection	Yes	(5); (6); (8)
Tumours	Yes	If otherwise healthy and no current chemotherapy: (3)
Diabetes mellitus	Yes	If well controlled: (3) If poorly controlled: (1) until improvement; alternatively: (2)
Chronic kidney disease	Yes	(7)
Asplenia	Yes	(3); In case of elective splenectomy: (9)
Long-term glucocorticoid therapy	Yes	If cat is on low-dose anti-inflammatory glucocorticoid treatment: (3) If cat is on high-dose or long-term immunosuppressive glucocorticoid treatment: (4); alternatively: (2)
Long-term cyclosporine therapy	Yes	Primary vaccination series: (4); alternatively: (2) Booster vaccination (if cat otherwise healthy): (3)
Chemotherapy for tumours	Yes	(4); alternatively: (2)
General anaesthesia/peri-operative period	Potentially ***	(1); if vaccination cannot be avoided (e.g., trap-neuter): (3)
Senior cats (>11 years)	Yes	Primary vaccination series: (10) Booster vaccination: (3)

* To avoid unnecessary vaccination against feline panleukopenia virus (FPV) in case antibody levels are adequate. ** Vaccination should be avoided even if no antibodies are present; therefore measuring FPV antibodies is not useful in this situation. *** Depending on the situation, antibody measurement might be useful in adult cats in this situation to avoid unnecessary vaccinations. (1) Postpone vaccination until recovered/end of treatment. (2) Consider administering passive immunisation (transfer of antibodies against FPV, feline calicivirus (FCV), and feline herpesvirus (FHV)) if available in case of high infectious pressure. (3) Vaccinate as for clinically healthy cats. (4) Postpone vaccination until at least three months after the end of treatment. (5) Keep retrovirus-infected cats strictly indoors. (6) Only consider vaccination if the cat is clinically healthy. (7) Base decision to vaccinate on risk-benefit ratio, e.g., avoid vaccinating indoor-only adult cats (unless the cat lacks FPV antibodies). (8) Consider annual booster of core vaccines. (9) Complete vaccination at least two weeks before surgery. (10) Give two injections of primary vaccination series at a three to four week interval (including MLV and rabies vaccines) OR perform FPV and rabies antibody measurement after the first injection to verify if protection is adequate.
